# Copper Ions Facilitate the Conjugative Transfer of SXT/R391 Integrative and Conjugative Element Across Bacterial Genera

**DOI:** 10.3389/fmicb.2020.616792

**Published:** 2021-02-02

**Authors:** Zhou Song, Lei Zuo, Cui Li, Yiming Tian, Hongning Wang

**Affiliations:** Animal Disease Prevention and Food Safety Key Laboratory of Sichuan Province, Key Laboratory of Bio-Resource and Eco-Environment of Ministry of Education, College of Life Sciences, Sichuan University, Chengdu, China

**Keywords:** copper ions, SXT/R391 integrative and conjugative element, conjugative transfer, reactive oxygen species, cell membrane permeability

## Abstract

Copper can persist stably in the environment for prolonged periods. Except for inducing antibiotic resistance in bacteria, copper ions (Cu^2+^) can facilitate the horizontal transfer of plasmid DNA. However, whether and how Cu^2+^ can accelerate the conjugative transfer of SXT/R391 integrative and conjugative element (ICE) is still largely unknown. In this study, *Proteus mirabilis* ChSC1905, harboring an SXT/R391 ICE that carried 21 antibiotic resistance genes (ARGs), was used as a donor, and *Escherichia coli* EC600 was used as a recipient. Cu^2+^, at subinhibitory and environmentally relevant concentrations (1–10 μmol/L), significantly accelerated the conjugative transfer of SXT/R391 ICE across bacterial genera (from *P. mirabilis* to *E. coli*) (*p* < 0.05). The combined analyses of phenotypic tests and genome-wide sequencing indicated that reactive oxygen species (ROS) production and cell membrane permeability were critical in the enhanced conjugative transfer of SXT/R391 ICE. Furthermore, the expression of genes related to cell adhesion and ATP synthesis was also significantly upregulated on exposure to Cu^2+^ at a concentration of 5 μmol/L. This study clarified the potential mechanisms of Cu^2+^ to promote the conjugative transfer of SXT/R391 ICE, revealing the potential risk imposed by Cu^2+^ on the horizontal transfer of SXT/R391 ICE-mediated ARGs.

## Introduction

Currently, the extensive use of heavy metal copper in plants, livestock, and hospitals is a serious threat to public health (Zhu et al., 2013; Pal et al., 2017). Besides, the residual copper persists stably in the environment for prolonged periods (Yu et al., 2017). Copper can induce antibiotic resistance by coselection (Li et al., 2017). For instance, copper can drive the development of antibiotic resistance (Poole, 2017) owing to the mobile genetic elements carrying both antibiotic resistance genes (ARGs) and metal resistance genes (Sandegren et al., 2012; Yang et al., 2018). On the contrary, horizontal gene transfer (HGT) is another critical driver for disseminating ARGs in various environments (Wang et al., 2015; Li et al., 2020). Copper ions (Cu^2+^) promote the horizontal transfer of plasmid-mediated ARGs in freshwater microcosms (Wang et al., 2020). HGT includes conjugation (mediated by cell-to-cell contact), transformation (mediated by extracellular DNA), and transduction (bacteriophage mediated) (Partridge et al., 2018; McInnes et al., 2020; Nihemaiti et al., 2020). The conjugation between the donor and the recipient mostly mediates the transfer of plasmids and integrative and conjugative elements (ICEs) (Hastings et al., 2004; Redondo-Salvo et al., 2020; Rodriguez-Beltran et al., 2020). In addition, the conjugative transfer of DNA may occur within or across bacterial genera (Thomas and Nielsen, 2005; Wang et al., 2019), leading to the dissemination of ARGs among a wide range of bacterial species (Shun-Mei et al., 2018).

Nevertheless, apart from the horizontal transfer of plasmid DNA (Xie et al., 2019), increasing evidence suggested that SXT/R391 ICEs were critical drivers for the spread of ARGs, harboring the integrase gene *int*, a marker to define SXT/R391 ICEs in clinical strains (Wozniak et al., 2009; Bioteau et al., 2018). Recently, many clinically important ARGs were found to be located on SXT/R391 ICEs (Aberkane et al., 2016), for example, carbapenemase gene *bla*_*NDM*–1_ (Kong et al., 2020), fosfomycin resistance gene *fosA3* (Lei et al., 2018), and tigecycline resistance gene *tet(X6)* (He et al., 2020). These SXT/R391 ICEs were also transferable (Badhai and Das, 2016). Antibiotics, such as ciprofloxacin, could induce SXT ICE transfer *via* SOS response (Beaber et al., 2004). However, studies investigating whether other non-antibiotic materials, especially Cu^2+^, can facilitate the conjugative transfer of SXT/R391 ICE across bacterial genera were limited. As reported, Cu^2+^ at subinhibitory concentrations could promote the horizontal transfer of RP4 plasmid in water environment (Zhang et al., 2018a), mainly *via* increased intracellular reactive oxygen species (ROS) generation, activated SOS response, and enhanced cell membrane permeability (Zhang et al., 2019). Therefore, it was hypothesized that Cu^2+^ could accelerate the conjugative transfer of SXT/R391 ICE in the same or a different way.

To confirm the aforementioned hypothesis, an SXT/R391 ICE that carried 21 ARGs was selected. Subsequently, the effects of Cu^2+^, at subinhibitory and environmentally relevant concentrations, on the conjugative transfer of this SXT/R391 ICE from *Proteus mirabilis* to *Escherichia coli* were assessed. Moreover, the mechanisms were explored by testing the intracellular ROS level, detecting cell membrane permeability, and checking changes in the expression of genes related to oxidative stress, SOS response, cell membrane, cell adhesion, and ATP synthesis. The present study was novel in exploring the effects and potential mechanisms of Cu^2+^ on the conjugative transfer of SXT/R391 ICE. The findings provided insights into the role of Cu^2+^ in the transfer of SXT/R391 ICE and emphasized the necessity for the proper use of copper in the future.

## Materials and Methods

### Bacterial Strains and Culture Conditions

*Proteus mirabilis* ChSC1905 (accession no. CP047929), isolated from the nasal swab of a diseased pig in China, was selected as the donor strain. The donor *P. mirabilis* harbored an SXT/R391 ICE that carried 21 ARGs, including a gene for resistance to cefotaxime (CTX). *E. coli* EC600, with higher resistance to rifampicin (RD), was chosen as the recipient strain. Both the donor and the recipient strains were incubated at 37°C in Luria broth (LB, Sigma, United States) medium with shaking at 180 rpm overnight. Then, the prepared bacterial strains were obtained by centrifuging (6,000 *g*) at 4°C for 5 min. After removing the supernatants, the pellets were washed with phosphate-buffered saline (PBS, Sangon, China) and resuspended in PBS. The CuSO_4_.5H_2_O solution was prepared with sterile water.

### Minimum Inhibitory Concentrations Test

The minimum inhibitory concentrations (MICs) of Cu^2+^, CTX, and RD against donor *P. mirabilis* ChSC1905 and recipient *E. coli* EC600 were measured as described previously (Fang et al., 2016; Lu et al., 2020). Briefly, overnight cultures of the donor *P. mirabilis* and the recipient *E. coli* were diluted to approximately 10^5^ cfu/ml. Then, 5 μl of the bacterial solution, 15 μl of Cu^2+^, CTX, or RD (at different concentrations), and 130 μl of fresh LB media were added. Sterilized LB was chosen as blank control. Subsequently, the 96-well plates were incubated at 37°C for 18 h, and then, a microplate spectrophotometer was used to obtain the optical density at 600 nm (OD600). MICs were obtained at the concentrations of Cu^2+^, CTX, and RD that completely inhibited the bacterial growth. All MIC tests were detected at least in triplicate.

### Conjugation Experiment Under Exposure of Cu^2+^

The conjugation mating system was established as described previously (Zhang et al., 2019). Briefly, 100 μl of the donor strain and the recipient strain were mixed at a 1:1 ratio with 10^8^ cfu/ml. Then, the mating system was exposed to different final concentrations of Cu^2+^ (0, 0.5, 1, 5, 10, and 100 μmol/L, total volume of 1 ml) in the presence of DNase I (a final concentration of 285 mg/L, Sigma). After incubating for 18 h without shaking at 37°C in PBS, 50 μl of the mixtures were inoculated on eosin methylene blue (EMB) agar selection plates containing 80 mg/L RD and 16 mg/L CTX for 48 h. EMB agar plates containing 80 mg/L RD were used to determine the total recipient numbers. The conjugative transfer frequency was calculated by dividing the total numbers of transconjugants by the total numbers of recipients. Furthermore, an ROS scavenger (100 mM thiourea) was added to the aforementioned conjugation mating systems to assess whether Cu^2+^ accelerated conjugative transfer through inducing ROS generation. The conjugation frequency in the thiourea-treated group was compared with that of the non-thiourea-treated group. All the conjugation experiments were conducted with biological triplicates. In parallel, a 10^8^ cfu/ml suspension of the donor and recipient strains were serially diluted of which 50 μl were plated onto EMB agar plates containing 80 mg/L RD and 16 mg/L CTX for 48 h. No colony indicating the emergence of spontaneous mutants could be observed at 10^8^ cfu/ml, indicating that there were no spontaneous mutants of the strains in this study. Additionally, an Enhanced Cell Counting Kit-8 (Beyotime, China) was used to count living cells in the presence of Cu^2+^ to determine whether Cu^2+^ influenced cell viability of the donor and recipient strains.

### Identification of Transconjugants

Transconjugants (five colonies selected randomly from each treatment group) were cultured overnight in LB media. The DNA of transconjugants was extracted using a Bacterial DNA Kit (Omega, United States) following the manufacturer’s protocols. Transconjugants were identified as *E. coli* using a BD PhoenixTM-100 Automated Microbiology System (Becton Dickinson, United States) (Kong et al., 2017) and 16S ribosomal DNA (rDNA) sequencing. The transconjugants were further defined by the antimicrobial resistance profile (Kirby–Bauer disk diffusion method based on the CLSI guidelines) (Clinical and Laboratory Standards Institute [CLSI], 2016). Finally, the *int* gene and the attachment sites *attL* and *attR* of SXT/R391 ICE in transconjugants were detected by polymerase chain reaction (Supplementary Table 1) to further confirm the presence of ICE in each transconjugant (Lei et al., 2016, 2018).

### Reverse Conjugation Experiment

The reverse conjugation mating system in the presence of Cu^2+^ at 5 μmol/L was further used to determine whether Cu^2+^ could still facilitate the transfer of SXT/R391 ICE from newly generated transconjugants to another recipient. The transconjugant (*E. coli* EC600 carrying SXT/R391 ICE) was used as a new donor. *E. coli* J53, with higher resistance to sodium azide, was selected as a new recipient. EMB agar plates containing 200 mg/L sodium azide and 16 mg/L CTX were used to select the transconjugants. The conjugative transfer frequency, spontaneous mutations of the strains, and identification of transconjugants were evaluated as described earlier.

### Detection of ROS and Cell Membrane Permeability

One hundred microliters of the donor *P. mirabilis* ChSC1905 (10^8^ cfu/ml) and the recipient *E. coli* EC600 (10^8^ cfu/ml) were separately exposed to different final concentrations of Cu^2+^ (0, 0.5, 1, 5, 10, and 100 μmol/L, total volume of 1 ml). For ROS detection, an ROS Assay Kit (Beyotime, China) was employed following the manufacturer’s protocols. Briefly, after incubating for 18 h, bacteria strains were incubated individually with 2′,7′-dichlorodihydrofluorescein diacetate (DCFH-DA, a final concentration of 10 μmol/L) for 20 min at 37°C. Then, samples were tested by the CytoFLEX flow cytometer (Beckman, United States) at 488 nm. For cell membrane permeability test, the strains were stained with 5 μl of propidium iodide (PI, Keygen, China) and incubated for 15 min in the dark before detecting by flow cytometer at 488 nm. All data were analyzed using CytExpert. All the tests were detected at least in triplicate.

### Transmission Electron Microscope

A transmission electron microscope (TEM) was employed to check whether the cell membrane or morphology was changed in the presence of Cu^2+^. Briefly, the mating system was exposed to Cu^2+^ at 5 and 0 μmol/L. After incubating for 18 h, the mixed samples were collected, fixed, dehydrated, filtered, and mounted. Ultrathin sections (50–100 nm) of each sample were applied on TEM copper grids and obtained using a JEM-1400PLUS TEM (Jeol, Japan) at 80 kV.

### Whole-Genome RNA Sequencing

The mating system was exposed to Cu^2+^ at 5 μmol/L (treatment group) and 0 μmol/L (control group). After mating for 18 h, the cells were collected, and the total RNA of each sample was extracted. Then, all samples (three samples from the control group and three samples from the treatment group) were submitted to Novogene (Beijing, China) for strand-specific complementary DNA (cDNA) library construction and NovaSeq 6000 (Illumina, United States) Illumina paired-end sequencing (clean bases, 2G). The reads containing adapter, poly-N, and low-quality reads were removed from the raw data. The obtained clean reads (each sample) were aligned to the *P. mirabilis* reference genome (NC_010554), *E. coli* reference genome (NC_000913) and the SXT sequence (MG773277) using Bowtie2 (v2.2.3). HTSeq (v0.6.1) was used to count the read numbers mapped to each gene. The gene expression was calculated as fragments per kilobase of a gene per million mapped reads (FPKM). Differential expression analysis (with biological replicates) was performed using the DESeq R package (v1.18.0). Differences in fold changes between 0 and 5 μmol/L Cu^2+^-treated mating system were calculated using log_2_ fold change (LFC) between control and Cu^2+^-treated samples. Genes with | LFC| > 0 and *p* < 0.05 were considered as differentially expressed. The visual images were analyzed using GraphPad Prism (v8.0.1).

### Statistical Analysis

Data were expressed as mean ± standard deviation. SPSS 19.0 (IBM, United States) was used for data analysis. An independent-samples *t* test was performed to analyze significant differences. *p* < 0.05 indicated a statistically significant difference (^∗^*p* < 0.05; ^∗∗^*p* < 0.01).

## Results

### Effects of Cu^2+^ on the Conjugative Transfer of SXT/R391 ICE

**FIGURE 1:**
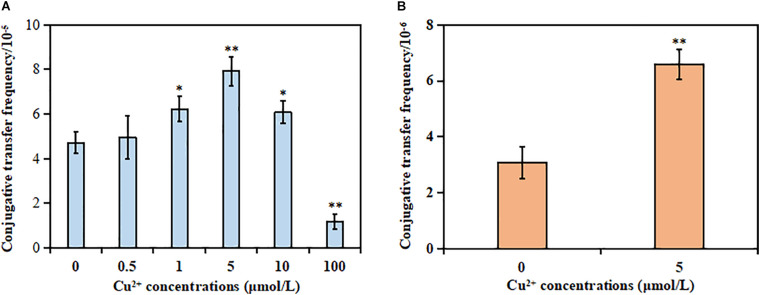
Effects of Cu^2+^ on the conjugative transfer of SXT/R391 integrative and conjugative element (ICE). **(A)** Conjugative transfer frequency of SXT/R391 ICE from the donor (*Proteus mirabilis* ChSC1905) to the recipient (*Escherichia coli* EC600). **(B)** Reverse conjugative transfer from the transconjugants (*E. coli* EC600) to *E. coli* J53. *p* < 0.05 indicated a statistically significant difference (*p < 0.05; **p < 0.01).

The results showed that the MIC of Cu^2+^, CTX, and RD against the donor strain was 10 mmol/L, >1,024 μg/ml, and 80 μg/ml, respectively. However, the MIC of Cu^2+^, CTX, and RD against the recipient strain was 10 mmol/L, <2 μg/ml, and 320 μg/ml, respectively. The mating system was exposed to different sub-MIC concentrations of Cu^2+^ to check whether Cu^2+^ could promote the conjugative transfer of SXT/R391 ICE. No spontaneous mutants were observed [frequency bellow 1/(5 × 10^6^ cells)]. As shown in Figure 1A, the conjugative transfer frequency of SXT/R391 ICE significantly increased from 6.06 × 10^–5^ to 7.92 × 10^–5^ in the presence of Cu^2+^ at 1 μmol/L (*p* < 0.05), 5 μmol/L (*p* < 0.01), and 10 μmol/L (*p* < 0.05) compared with the control (4.70 × 10^–5^), respectively. Particularly, the conjugative transfer frequency displayed a maximum increase at 5 μmol/L Cu^2+^. On the contrary, the conjugative transfer frequency (1.17 × 10^–5^) on exposure to Cu^2+^ at 100 μmol/L significantly decreased compared with the control (*p* < 0.01). This was likely due to the reduced cell viability of the donor and recipient strains (Supplementary Figure 2).

Various analyses were performed to verify that the SXT/R391 ICE was transferred successfully from the donor *P. mirabilis* to the recipient *E. coli*. First, the transconjugants were identified as *E. coli* species. Second, the donor *P. mirabilis* exhibited multidrug resistance besides being intrinsically resistant to colistin and tetracycline (Lei et al., 2018; Supplementary Table 2). However, the recipient *E. coli* was resistant only to rifampin. Indeed, the transconjugants displayed the same antimicrobial resistance profile compared with the donor, including resistance to cefotaxime, ceftriaxone, florfenicol, gentamicin, trimethoprim/sulfamethoxazole, fosfomycin, ampicillin, nalidixic acid, ciprofloxacin, amikacin, linezolid, and rifampin (Supplementary Table 2). Finally, both the donor strain and the transconjugants harbored the *int* gene, but the recipient strain did not. Besides, the attachment sites *attL* and *attR* of SXT/R391 ICE in transconjugants were detected (Supplementary Figure 1), further indicating that the transconjugants carried the SXT/R391 ICE from the donor.

The reverse conjugation experiment showed that the SXT/R391 ICE could be transferred from transconjugant (*E. coli* EC600) to another recipient (*E. coli* J53). Besides, no spontaneous mutants were observed [frequency bellow 1/(5 × 10^6^ cells)]. The conjugative frequency (6.59 × 10^–6^) in the presence of Cu^2+^ at 5 μmol/L also significantly increased compared with the control (3.08 × 10^–6^; *p* < 0.01; Figure 1B). The azide resistance of the auxotrophic mutant strain *E. coli* J53 was due to a single nucleotide substitution in the *secA* gene, whose genome contained a large inversion and missed five prophage regions and 18 non-hypothetical genes (Yi et al., 2012). However, *E. coli* EC600 was a bacteriophage λ-sensitive strain, the genome of which contained six prophage-associated regions (Allué-Guardia et al., 2019). Although both the two strains were derived from progenitor strain K-12, the aspect of strain J53 was distinguishable from strain EC600 on EMB selection plates. After excluding the occurrence of spontaneous mutations of the two strains, the transconjugants in the reverse conjugation experiment could be identified as *E. coli* J53 carrying the SXT/R391 ICE.

### Effects of Cu^2+^ on ROS Production

**FIGURE 2:**
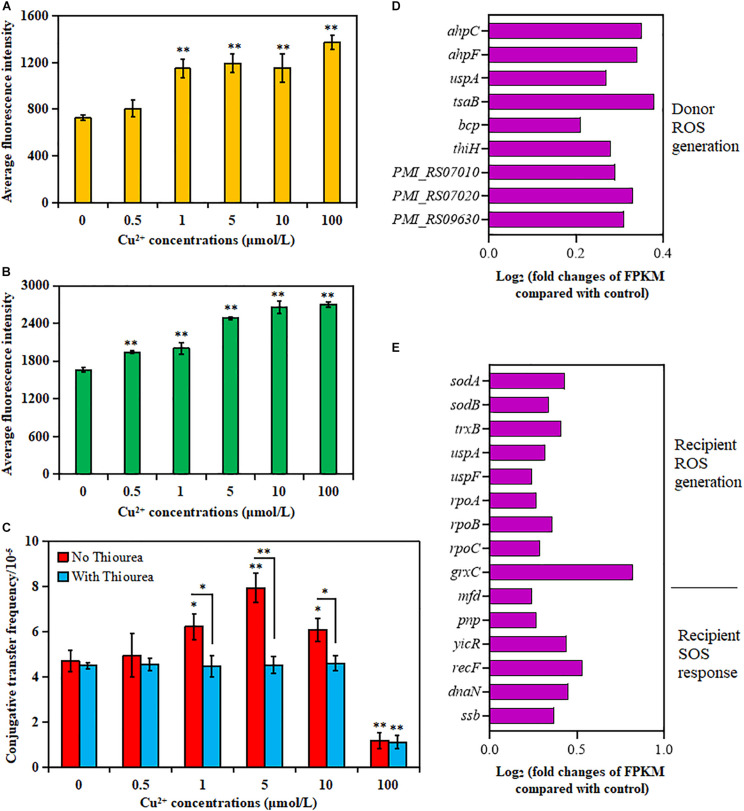
Effects of Cu^2+^ on reactive oxygen species (ROS) production in the donor (*Proteus mirabilis* ChSC1905) and recipient (*Escherichia coli* EC600). Average fluorescence intensity of ROS production in **(A)** the donor and **(B)** the recipient checked by flow cytometer. **(C)** Effect of ROS scavenger (thiourea) on the conjugative transfer of SXT/R391 integrative and conjugative element (ICE) from the donor to the recipient. Fold changes of the expression of genes related to ROS generation and SOS response in **(D)** the donor and **(E)** the recipient. *p* < 0.05 indicated a statistically significant difference (*p < 0.05; **p < 0.01).

As shown in Figure 2A, the donor strain displayed a significant increase in ROS generation in the presence of Cu^2+^ from 1 to 100 μmol/L compared with the control (*p* < 0.01). Similarly, the ROS generation also significantly increased for the recipient strain in the presence of Cu^2+^ from 0.5 to 100 μmol/L (*p* < 0.01; Figure 2B). The maximum value was obtained at 100 μmol/L Cu^2+^.

The conjugative transfer frequency decreased significantly in the presence of Cu^2+^ at 1 μmol/L (*p* < 0.05), 5 μmol/L (*p* < 0.01), and 10 μmol/L (*p* < 0.05) on adding ROS scavenger thiourea (Figure 2C). Nevertheless, the conjugative transfer frequency showed no significant difference in the presence of Cu^2+^ at 0, 0.5, and 100 μmol/L (*p* > 0.05). The results indicated that Cu^2+^ might promote the conjugative transfer of SXT/R391 ICE by inducing ROS production.

The changes in gene expression also supported the aforementioned phenotypes (Supplementary Tables 3, 4). As shown in Figures 2D,E, the cellular antioxidant-related genes were overexpressed on exposure to Cu^2+^ at 5 μmol/L, including genes coding for alkyl hydroperoxide reductase (*ahpC* and *ahpF*) in the donor strain, as well as genes coding for superoxide dismutase (*sodA* and *sodB*) and thioredoxin reductase (*trxB*) in the recipient strain (Hayden et al., 2018). The expression of RNA polymerase (*rpoA*, *rpoB*, and *rpoC* genes) in the recipient strain was also upregulated, which was related to the induction of ROS production (Piccaro et al., 2014). The expression of the *uspA* gene, related to the survival of bacteria (Bandyopadhyay and Mukherjee, 2020), also increased in both donor and recipient strains. Besides, the expression of genes related to the ROS response in the donor strain (*tsaB*, *bcp*, *thiH*, *PMI_RS07010*, *PMI_RS07020*, and *PMI_RS09630*) and the recipient strain (*uspF* and *grxC*) also increased significantly. However, the genes related to the SOS response were mainly enriched in DNA repair and recombination (Figure 2E and Supplementary Table 4) in the recipient strain, including the enhanced expression of *mfd*, *ssb*, *yicR*, *recF*, *dnaN*, and *pnp* (Fan et al., 2019).

### Effects of Cu^2+^ on Cell Membrane Permeability

**FIGURE 3:**
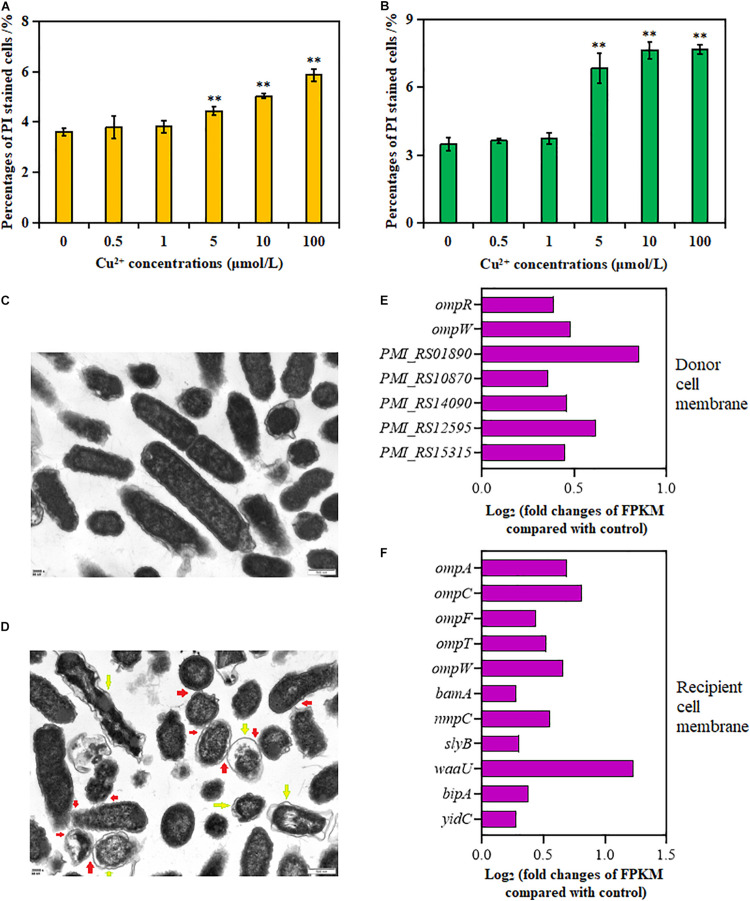
Effects of Cu^2+^ on cell membrane permeability in the donor (*Proteus mirabilis* ChSC1905) and recipient (*Escherichia coli* EC600). Percentages of PI-stained cells in **(A)** the donor and **(B)** recipient checked by flow cytometer. **(C)** Transmission electron microscopy (TEM) images in ultrafine slices of the control. **(D)** Cells exposure to Cu^2+^ at 5 μmol/L (scale bars, 500 nm). Yellow arrows stand for membrane damage; red arrows stand for cell-to-cell contact. Fold changes of the expression of genes related to cell membrane in the **(E)** donor and **(F)** recipient. *p* < 0.05 indicated a statistically significant difference (*p < 0.05; **p < 0.01).

As shown in Figures 3A,B, the percentage of PI-positive cells in both donor and recipient strains significantly increased in the presence of Cu^2+^ at 5, 10, and 100 μmol/L (*p* < 0.01); the maximum value was obtained at 100 μmol/L Cu^2+^. These results showed that Cu^2+^ enhanced cell membrane permeability.

Transmission electron microscope images of the cell morphology and membrane showed dispersed cells with less physical contact, distinct cell membranes, and compact cytoplasm in the control group (Figure 3C). On exposure to Cu^2+^ at 5 μmol/L (Figure 3D), more cell adhesion between these cells was observed. Besides, apparent cell membrane damage and indistinct cell borders were found.

The changes in gene expression were also consistent with the aforementioned cell membrane phenotypes (Supplementary Tables 5, 6). As shown in Figures 3E,F, the expression of genes in the donor strain encoding for membrane proteins, *omp* gene family (*ompR* and *ompW*), *PMI_RS01890*, *PMI_RS10870*, *PMI_RS14090*, *PMI_RS12595*, and *PMI_RS15315*, was significantly upregulated on exposure to Cu^2+^ at 5 μmol/L. Similarly, in the recipient strain, in addition to *omp* gene family (*ompA*, *ompC*, *ompF*, *ompT*, and *ompW*), the expression of lipopolysaccharide synthesis gene *waa*, gene *bamA* coding for outer membrane assembly protein, gene *slyB* coding for outer membrane lipoprotein (Lu et al., 2020), gene *nmpC* coding for outer membrane porin (Ruan et al., 2011), gene *yidC* involved in insertion and folding of membrane proteins (Gray et al., 2011), and gene *bipA* was also significantly upregulated.

### Effects of Cu^2+^ on Cell Adhesion and ATP Synthesis

**FIGURE 4:**
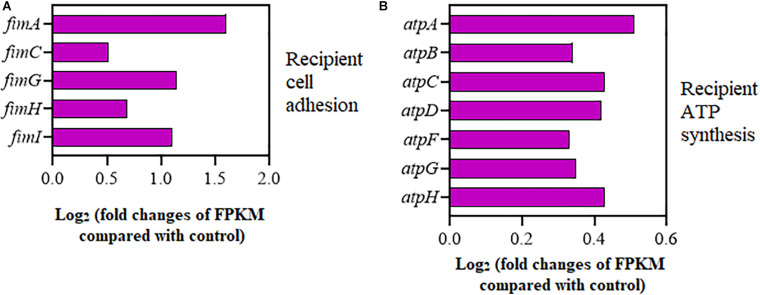
Effects of Cu^2+^ on cell adhesion and ATP synthesis in the recipient (*Escherichia coli* EC600). Fold changes of the expression of genes related to **(A)** cell adhesion and **(B)** ATP synthesis.

As shown in Figure 4A, the expression of adhesion-relevant genes *fimA*, *fimC*, *fimG*, *fimH*, and *fimI* was significantly upregulated in the recipient strain on exposure to Cu^2+^ at 5 μmol/L (Supplementary Table 7). Additionally, the expression of seven ATP encoding genes, *atpA*, *atpB*, *atpC*, *atpD*, *atpF*, *atpG*, and *atpH*, which controlled cellular energy production, was also significantly increased in the recipient strain (Figure 4B and Supplementary Table 8). Interestingly, the expression of the *copA* gene encoding for Cu^+^ translocating P-type ATPase was also significantly upregulated in the recipient strain (Supplementary Table 8).

## Discussion

Except for the antibiotic-driven spread of ARGs (Blazquez et al., 2012; Lopatkin et al., 2016; Liu et al., 2017; Jutkina et al., 2018), non-antibiotic materials also accelerated the dissemination of plasmid-mediated ARGs (Qiu et al., 2012; Jiao et al., 2017; Zhang et al., 2018b; Cen et al., 2020). In particular, previous studies demonstrated that Cu^2+^ could promote the conjugative transfer of plasmid DNA within bacterial genera (from *E. coli* S17-1 to *E. coli* K12 MG1655) (Zhang et al., 2018a) or across bacterial genera (from *E. coli* K-12 LE392 to *P. putida* KT2440) (Zhang et al., 2019). However, whether Cu^2+^ could facilitate the conjugative transfer of SXT/R391 ICE was rarely explored. The present study showed that the conjugative transfer of SXT/R391 ICE that carried a large number of ARGs across bacterial genera (from *P. mirabilis* to *E. coli*) could be significantly promoted by Cu^2+^ ranging from 1 to 10 μmol/L (Figure 1A). Noticeably, subinhibitory and environmentally relevant concentrations of Cu^2+^ were used in this study (Zhang et al., 2019). The antimicrobial resistance profile, the *int* gene, and the attachment sites *attL* and *attR* of SXT/R391 ICE in transconjugants were detected, implying that the transconjugants carried the SXT/R391 ICE from the donor. Further, 5 μmol/L Cu^2+^ significantly facilitated the conjugative transfer of SXT/R391 ICE from the newly generated transconjugants to another recipient (Figure 1B), indicating that these newly generated transconjugants might serve as a novel ARG source at low concentrations of Cu^2+^. These findings confirmed the viewpoint that Cu^2+^ could accelerate the conjugative transfer of SXT/R391 ICE.

The addition of ROS scavenger significantly decreased the conjugative frequency (Figure 2C), suggesting that the increased production of ROS in both donor and recipient strains (Figures 2A,B) was caused by Cu^2+^ and was crucial for the transfer of SXT/R391 ICE. The bacterial cells rapidly respond to oxidative stress to protect against ROS attack (Liao et al., 2019). As expected, in the present study (Figures 2D,E), the expression of antioxidant-related genes, such as *ahpC* and *ahpF*, in the donor strain and *sodA*, *sodB*, and *trxB* in the recipient strain, was upregulated on exposure to Cu^2+^ at 5 μmol/L. These antioxidant enzymes were probably expressed to protect the donor and recipient strains from the ROS attack (Zuo et al., 2014; Zhu et al., 2019) due to increased ROS generation. Therefore, it was considered that the change in ROS generation was a critical factor for Cu^2+^ to promote the conjugative transfer of SXT/R391 ICE. However, ROS overproduction might cause irreversible cell function damage and even cell death, making recipient cells inactive. A previous report showed that Cu^2+^ and CuO nanoparticles at 100 μmol/L could decrease the horizontal transfer of plasmid-mediated ARGs due to the reduced recipient number (Zhang et al., 2019). Higher sub-MIC concentrations of Cu^2+^ reduced conjugative transfer of plasmids (Buberg et al., 2020). As reported, 100 μmol/L Cu^2+^ suppressed the transfer of plasmid-mediated ARG in a sludge bacterial community, probably attributed to disrupted iron–sulfur clusters of metalloenzymes and the purified fumarase A poisoning (Lin et al., 2019). Besides, 100 μmol/L Cu^2+^ significantly reduced the cell viability of the donor and recipient strains compared with the control (Supplementary Figure 2). Thus, these might be the reasons for the decreased transfer frequency of SXT/R391 ICE on exposure to Cu^2+^ at 100 μmol/L in this study.

Reactive oxygen species generation resulted in DNA damage, thus inducing the SOS response that controlled a series of genes involved in DNA damage repair and recombination (Baharoglu et al., 2010; Baharoglu and Mazel, 2014). In this study, the differentially expressed genes related to SOS response were mainly enriched in DNA repair and recombination in the recipient *E. coli* when exposed to Cu^2+^ at 5 μmol/L (Figure 2E). The SOS response could transitorily contribute to maintain a lower pool of SetR protein (an SXT-encoded repressor), thereby increasing the expression of genes necessary for SXT transfer (Beaber et al., 2004). This included activation of the site-specific recombination system, assembly of the mating apparatus, initiation of ICE DNA transfer, and integration into chromosome of recipient cell (Poulin-Laprade et al., 2015). However, there were no significant differences in the expression of genes related to the SOS response in the donor *P. mirabilis*, as well as the expression of genes related to SXT/R391 ICE transfer under exposure of 5 μmol/L Cu^2+^ in this study. Strain genetic background, insufficient sequencing coverage to detect rare transcripts, and extended mating periods may be contributing factors to the failure of SOS response and transfer genes transcripts detection.

Increased ROS production can cause damage to the cell membrane for both the donor and recipient strains, leading to an impaired membrane barrier (Lu et al., 2018). This was consistent with the TEM images, showing that apparent cell membrane damage and indistinct cell borders were found on exposure to Cu^2+^ at 5 μmol/L (Figure 3D). Moreover, ROS generation was known to enhance cell membrane permeability (Liao et al., 2019), which was associated with increased conjugative transfer frequency (Zhang et al., 2018a). Indeed, the cell membrane permeability of the donor and recipient strains increased significantly in the presence of Cu^2+^ (Figures 3A,B). The outer membrane proteins (e.g., OmpA, OmpC, and OmpF) played important roles in forming outer membrane pores and increasing membrane permeability (Zhang et al., 2017). The overexpression of outer membrane proteins accelerated the inward or outward movement of DNA (Dreiseikelmann, 1994). The transcriptional analyses suggested that the expression of relevant genes (e.g., *omp*) coding for outer membrane proteins was also upregulated on exposure to Cu^2+^ at 5 μmol/L (Figures 3E,F). Therefore, it was presumed that the increased membrane permeability was also a pivotal factor for Cu^2+^ to accelerate the transfer of SXT/R391 ICE.

Physical cell-to-cell contact is essential for the plasmid DNA transfer during the conjugative process. For example, *fim*-like operon has been reported to be related to adhesion (Wang et al., 2019). In this study, the expression of adhesion-relevant genes (e.g., *fim*) was upregulated on exposure to Cu^2+^ at 5 μmol/L in the recipient strain (Figure 4A), which was consistent with the TEM images (Figure 3D). These findings indicated that enhanced cell adhesion might contribute to the increased transfer of SXT/R391 ICE. Besides, increased messenger RNA (mRNA) expression of ATP synthesis genes (*atp*) was also observed in the recipient strain (Figure 4B). As DNA movement needs energy (Lu et al., 2018), improved energy availability may be contribute to the elevated transfer of SXT/R391 ICE *via* providing more energy. Interestingly, the expression of *copA* gene (a component of the copper efflux system) was upregulated in the recipient *E. coli* (Supplementary Table 8). The increased expression of gene *copA* might contribute to the recipient *E. coli* to avoid any excess copper-mediated toxicity and retain adequate supply of copper for cellular processes (Pal et al., 2017). As reported, enhancement of the K^+^ and Na^+^ efflux might promote the formation of transfer channel and plasmid uptake (Liao et al., 2019). Thus, the upregulated *copA* gene may be involved in the uptake of SXT/R391 ICE in this study.

## Conclusion

This study demonstrated that the conjugative transfer of SXT/R391 ICE across bacterial genera (from *P. mirabilis* to *E. coli*) could be significantly accelerated by Cu^2+^. Importantly, the SXT/R391 ICE we selected carried 21 ARGs and mediated multidrug resistance. ROS generation, cell membrane permeability, cell adhesion, and ATP synthesis were the potential mechanisms for Cu^2+^ to promote the conjugative transfer of SXT/R391 ICE. This study was novel in proving that Cu^2+^ could facilitate the conjugative transfer of SXT/R391 ICE at subinhibitory and environmentally relevant concentrations.

## Data Availability Statement

The datasets presented in this study can be found in online repositories. The names of the repository/repositories and accession number(s) can be found in the article/Supplementary Material.

## Ethics Statement

The animal study was reviewed and approved by Sichuan University Animal Ethics Committee.

## Author Contributions

ZS and HW conceived and designed the study. ZS, LZ, CL, and YT performed the experiments. ZS analyzed the data and wrote the manuscript. All authors contributed to the manuscript revision and approved the final manuscript.

## Conflict of Interest

The authors declare that the research was conducted in the absence of any commercial or financial relationships that could be construed as a potential conflict of interest.
