# Supplementary material for: Copper Ions Facilitate the Conjugative Transfer of SXT/R391 Integrative and Conjugative Element Across Bacterial Genera
Source: Front Microbiol. 2021 Feb 2;11:616792. doi: 10.3389/fmicb.2020.616792 (PMC7884315; doi:10.3389/fmicb.2020.616792)
Supplement: Supplementary file 1 [file Data_Sheet_1.doc]

**Supplementary Table S1.** Primers used for detecting the *int* gene and the attachment sites *attL* and *attR* of SXT/R391 ICE in transconjugants.

| Primers | Nucleotide sequences (5’-3’) | Targeted region | Length of product |
| --- | --- | --- | --- |
| int-F | CTGTGGCCAATCATCAACTC | SXT/R391 family *int* | 1035 bp |
| int-R | CGACCGAGATGGGCTAAGTG |
| LE1 | ACAACGATAACAGAGCATTGG | *attL* | 413 bp |
| LE4 | GTACACACTTTCCGAGGTTACG |
| RE1 | TGCACGTTGGATAGCTTGTCCG | *attR* | 466 bp |
| RE4 | CCGCAATACCCTGCAATACCGA |

**Supplementary Table S2.** Antimicrobial resistance profiles of the donor *P. mirabilis* ChSC1905, recipient *E. coli* EC600, and transconjugants (mediated by different concentrations of Cu^2+^).

| Strains | | Antimicrobial resistance profile |
| --- | --- | --- |
| *P. mirabilis* ChSC1905 | | PB-CTX-CRO-FFC-CN-SXT-FOS-AMP-NA-CIP-AK-LZD-RD-DO-TE |
| *E. coli* EC600 | | RD |
| Transconjugants | 0 (μmol/L) | CTX-CRO-FFC-CN-SXT-FOS-AMP-NA-CIP-AK-LZD-RD-DO-TE |
|  | 0.5 (μmol/L) | CTX-CRO-FFC-CN-SXT-FOS-AMP-NA-CIP-AK-LZD-RD-DO-TE |
|  | 1 (μmol/L) | CTX-CRO-FFC-CN-SXT-FOS-AMP-NA-CIP-AK-LZD-RD-DO-TE |
|  | 5 (μmol/L) | CTX-CRO-FFC-CN-SXT-FOS-AMP-NA-CIP-AK-LZD-RD-DO-TE |
|  | 10 (μmol/L) | CTX-CRO-FFC-CN-SXT-FOS-AMP-NA-CIP-AK-LZD-RD-DO-TE |
|  | 100 (μmol/L) | CTX-CRO-FFC-CN-SXT-FOS-AMP-NA-CIP-AK-LZD-RD-DO-TE |

PB, colistin; CTX, cefotaxime; CRO, ceftriaxone; FFC, florfenicol; CN, gentamicin; SXT, trimethoprim/sulfamethoxazole; FOS, fosfomycin; AMP, ampicillin; NA, nalidixic acid; CIP, ciprofloxacin; AK, amikacin; LZD, linezolid; RD, rifampin; DO, doxycycline; TE, tetracycline.

**Supplementary Table S3.** Genes related to ROS production in donor *P. mirabilis* ChSC1905 after exposure of Cu^2+^ at 5 μmol/L.

| Gene | Gene description | Log_2_ (fold change of FPKM)* |
| --- | --- | --- |
| *ahpC* | Alkyl hydroperoxide reductase subunit C | 0.35 |
| *ahpF* | Alkyl hydroperoxide reductase subunit F | 0.34 |
| *uspA* | Universal stress protein UspA | 0.27 |
| *tsaB* | tRNA (adenosine(37)-N6)-threonylcarbamoyltransferase complex dimerization subunit type 1 TsaB | 0.38 |
| *bcp* | Thioredoxin-dependent thiol peroxidase | 0.21 |
| *thiH* | 2-iminoacetate synthase ThiH | 0.28 |
| *PMI_RS07010* | Universal stress protein | 0.29 |
| *PMI_RS07020* | Universal stress protein | 0.33 |
| *PMI_RS09630* | Universal stress protein | 0.31 |

* (*p* < 0.05): Compared with the control group without Cu^2+^ treatment.

**Supplementary Table S4.** Genes related to ROS production and SOS response in recipient *E. coli* EC600 after exposure of Cu^2+^ at 5 μmol/L.

| Gene | Gene description | Log_2_ (fold change of FPKM)* |
| --- | --- | --- |
| *sodA* | Superoxide dismutase (Mn) | 0.43 |
| *sodB* | Superoxide dismutase (Fe) | 0.34 |
| *trxB* | Thioredoxin reductase | 0.41 |
| *uspA* | Universal stress global stress response regulator | 0.32 |
| *uspF* | Nucleotide binding filament protein | 0.24 |
| *rpoA* | RNA polymerase subunit alpha | 0.27 |
| *rpoB* | RNA polymerase subunit beta | 0.36 |
| *rpoC* | RNA polymerase subunit beta' | 0.29 |
| *grxC* | Glutaredoxin 3 | 0.82 |
| *mfd* | Transcription-repair coupling factor | 0.24 |
| *pnp* | Polynucleotide phosphorylase | 0.27 |
| *yicR* | RadC-like JAB domain-containing protein YicR | 0.44 |
| *recF* | DNA repair protein RecF | 0.53 |
| *dnaN* | Beta sliding clamp | 0.45 |
| *ssb* | ssDNA-binding protein | 0.37 |

* (*p* < 0.05): Compared with the control group without Cu^2+^ treatment.

**Supplementary Table S5.** Genes related to cell membrane in donor *P. mirabilis* ChSC1905 after exposure of Cu^2+^ at 5 μmol/L.

| Gene | Gene description | Log_2_ (fold change of FPKM)* |
| --- | --- | --- |
| *ompR* | Two-component system response regulator OmpR | 0.39 |
| *ompW* | Outer membrane protein OmpW | 0.48 |
| *PMI_RS01890* | Anaerobic c4-dicarboxylate membrane transporter | 0.85 |
| *PMI_RS10870* | Sodium:sulfate symporter transmembrane region | 0.36 |
| *PMI_RS14090* | Predicted permease membrane region | 0.46 |
| *PMI_RS12595* | Anaerobic c4-dicarboxylate membrane transporter | 0.62 |
| *PMI_RS15315* | Rhomboid family intramembrane serine protease | 0.45 |

* (*p* < 0.05): Compared with the control group without Cu^2+^ treatment.

**Supplementary Table S6.** Genes related to cell membrane in recipient *E. coli* EC600 after exposure of Cu^2+^ at 5 μmol/L.

| Gene | Gene description | Log_2_ (fold change of FPKM)* |
| --- | --- | --- |
| *ompA* | Outer membrane porin A | 0.69 |
| *ompC* | Outer membrane porin C | 0.81 |
| *ompF* | Outer membrane porin F | 0.44 |
| *ompT* | Outer membrane protease VII (outer membrane protein 3b) | 0.52 |
| *ompW* | Outer membrane protein W | 0.66 |
| *bamA* | Outer membrane protein assembly factor BamA | 0.28 |
| *nmpC* | DLP12 prophage%3B putative outer membrane porin NmpC | 0.55 |
| *slyB* | Outer membrane lipoprotein SlyB | 0.30 |
| *waaU* | Putative ADP-heptose:LPS heptosyltransferase 4 | 1.23 |
| *bipA* | Ribosome-dependent GTPase%2C ribosome assembly factor | 0.38 |
| *yidC* | Membrane protein insertase YidC | 0.28 |

* (*p* < 0.05): Compared with the control group without Cu^2+^ treatment.

**Supplementary Table S7.** Genes related to cell adhesion in recipient *E. coli* EC600 after exposure of Cu^2+^ at 5 μmol/L.

| Gene | Gene description | Log_2_ (fold change of FPKM)* |
| --- | --- | --- |
| *fimA* | Type 1 fimbriae major subunit | 1.60 |
| *fimC* | Type 1 fimbriae periplasmic chaperone | 0.51 |
| *fimG* | Type 1 fimbriae minor subunit FimG | 1.14 |
| *fimH* | Type 1 fimbriae D-mannose specific adhesin | 0.69 |
| *fimI* | Putative fimbrial protein FimI | 1.10 |

* (*p* < 0.05): Compared with the control group without Cu^2+^ treatment.

**Supplementary Table S8.** Genes related to ATP synthesis in recipient *E. coli* EC600 after exposure of Cu^2+^ at 5 μmol/L.

| Gene | Gene description | Log_2_ (fold change of FPKM)* |
| --- | --- | --- |
| *atpA* | ATP synthase F1 complex subunit alpha | 0.51 |
| *atpB* | ATP synthase Fo complex subunit a | 0.34 |
| *atpC* | ATP synthase F1 complex subunit epsilon | 0.43 |
| *atpD* | ATP synthase F1 complex subunit beta | 0.42 |
| *atpF* | ATP synthase Fo complex subunit b | 0.33 |
| *atpG* | ATP synthase F1 complex subunit gamma | 0.35 |
| *atpH* | ATP synthase F1 complex subunit delta | 0.43 |
| *copA* | Cu(+) exporting P-type ATPase | 1.68 |

* (*p* < 0.05): Compared with the control group without Cu^2+^ treatment.
