# Supplementary material for: Copper Ions Facilitate the Conjugative Transfer of SXT/R391 Integrative and Conjugative Element Across Bacterial Genera
Source: Front Microbiol. 2021 Feb 2;11:616792. doi: 10.3389/fmicb.2020.616792 (PMC7884315; doi:10.3389/fmicb.2020.616792)
Supplement: Supplementary file 2 [file Data_Sheet_2.pdf]

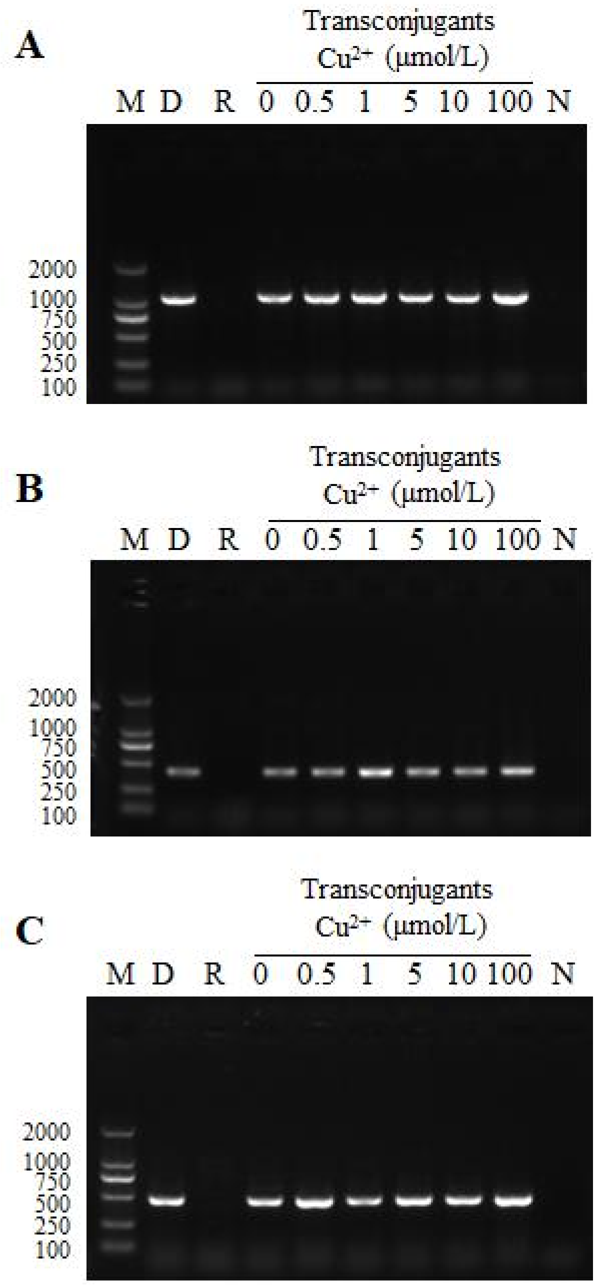

Supplementary Figure S1. Detection of the *int* gene and the attachment sites *attL* and *attR* of SXT/R391 ICE in transconjugants. **(A)** Detection of the *int* gene; **(B)** Detection of the attachment site *attL* of SXT/R391 ICE; **(C)** Detection of the attachment site *attR* of SXT/R391 ICE. M, marker DL2000; D, donor *P. mirabilis* ChSC1905; R, recipient *E. coli* EC600; N, normal.

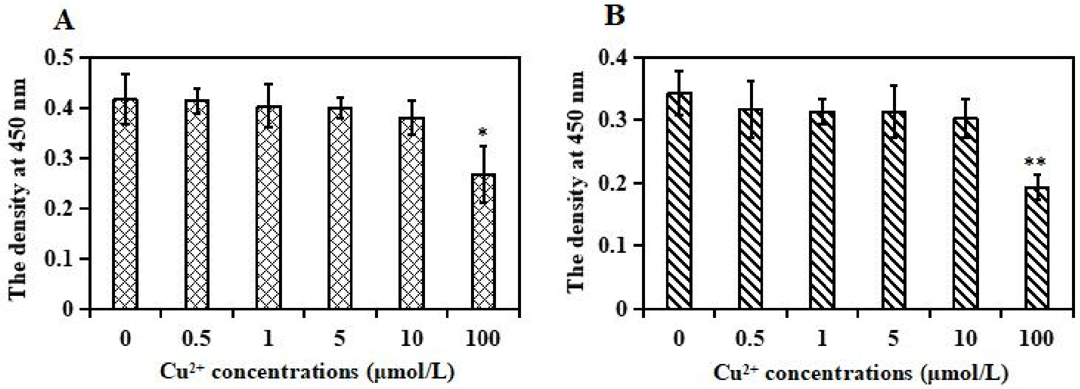

Supplementary Figure S2. Effects of Cu^{2+} on cell viability of the donor *P. mirabilis* ChSC1905 **(A)** and recipient *E. coli* EC600 **(B)**. An independent-samples *t* test was performed to analyze significant differences between Cu^{2+}-treated groups and control. $p < 0.05$ indicated a statistically significant difference (*$p < 0.05$; **$p < 0.01$). 100 μmol/L Cu^{2+} significantly reduced the cell viability of donor and recipient strains.
